# Non-target Effects of Naphthalene on the Soil Microbial Biomass and Bacterial Communities in the Subalpine Forests of Western China

**DOI:** 10.1038/s41598-019-46394-3

**Published:** 2019-07-08

**Authors:** Liying Lan, Fan Yang, Li Zhang, Wanqin Yang, Fuzhong Wu, Zhenfeng Xu, Yang Liu, Kai Yue, Xiangyin Ni, Han Li, Shu Liao, Yuwei Liu, Ya Chen, Bo Tan

**Affiliations:** 1 Institute of Ecology & Forestry, Sichuan Agricultural University, Forestry Ecological Engineering in Upper Reaches of Yangtze River Key Laboratory of Sichuan Province, Alpine Forest Ecosystem Research Station, Soil and Water Conservation and Desertification Control Key Laboratory of Sichuan Province, Chengdu, 611130 China; 2 Collaborative Innovation Center of Ecological Security in the Upper Reaches of Yangtze River, Chengdu, 611130 China

**Keywords:** Forest ecology, Forestry

## Abstract

Naphthalene is a biocide of soil fauna, particularly of soil arthropods, that has been widely applied to test the functional roles of soil fauna in soil processes. However, whether the use of naphthalene to expel soil fauna has a non-target effect on soil bacteria in subalpine forests remains unclear. We conducted a naphthalene treatment experiment to explore the effects of naphthalene on the soil bacterial community in subalpine forest soil. The results suggested that naphthalene treatment (at 100 g.m^−2^ per month) significantly increased the abundances of total bacterial, gram-positive bacterial and gram-negative bacterial phospholipid fatty acids (PLFA) and did not change the microbial biomass carbon (MBC), microbial biomass nitrogen (MBN) or MBC/MBN ratio. Moreover, a total of 1038 operational taxonomic units (OTUs) were detected by Illumina MiSeq sequencing analysis. Proteobacteria, Actinobacteria, and Acidobacteria Chloroflexi were the dominant phyla, and *Bradyrhizobium* was the most abundant genus. The naphthalene treatment did not affect soil bacterial diversity or community structure. Overall, these results demonstrated that the naphthalene treatment had non-target effects on the active bacterial community abundance but not the soil bacterial community structure. Thus, the non-target effects of naphthalene treatment should be considered before using it to expel soil fauna.

## Introduction

Soil microorganisms are the foundation of complex soil food webs, and these microorganisms participate in soil processes, such as litter decomposition, nutrient mineralization and greenhouse gas emissions, through the detrital food chain in forests^1,2^. Although soil microorganisms are the dominant drivers of most of these processes, soil fauna have been suggested to play a functional role in soil carbon and nutrient cycling by interacting with the microbial community^3–5^. Soil fauna can directly influence the quantity, activity, composition and function of soil microorganisms by selective feeding^6,7^ and can indirectly influence soil microorganisms by changing the soil microenvironment and decomposing litter^8^. Thus, the interactions between soil fauna and microorganisms should be studied to understand the mechanisms of material circulation and energy conversion on the forest surface^9^.

Quantifying the impacts of soil fauna on soil microorganisms is challenging because experiments are difficult to set up without affecting the microclimate and non-target species^10^. At present, researchers physically or chemically remove soil fauna to study the contribution of soil fauna to litter decomposition, nutrient release and other soil biogeochemical cycles^6,10–12^. However, physical methods (e.g., the litterbag method) cannot accurately determine the soil fauna’s contribution to microclimatic changes in the litterbags or to biological activity^11,12^. Chemical applications can be used to suppress certain biotic groups in order to confirm their contributions to soil biogeochemistry. For example, naphthalene has been used for decades in decomposition experiments in different regions to suppress soil fauna^13,14^. Previous studies have shown that naphthalene can directly inhibit soil fauna, such as soil arthropods^13–15^. Moreover, studies have also indicated that naphthalene may indirectly influence soil process through potential non-target effects on the microbial community and soil nutrients^16–18^. Therefore, it is necessary to study the non-target effects of naphthalene on soil microbial biomass, active bacterial abundance and the bacterial community when expelling soil fauna.

Subalpine forests constitute the main part of the southwestern forests of China, and rigorous field assessments of the efficacy of naphthalene treatment for suppressing soil fauna as well as its potential non-target effects on the soil microbial community are lacking in this region. We conducted research in a subalpine forest in western China by adding naphthalene to the soil surface. The study’s objectives were to (i) quantify naphthalene’s non-target effect on soil microbial biomass; (ii) measure naphthalene’s non-target effect on active bacterial abundance; and (iii) determine naphthalene’s non-target effect on the soil bacterial community structure.

## Results

### Soil microbial biomass and phospholipid fatty acids (PLFA)

**Figure 1:**
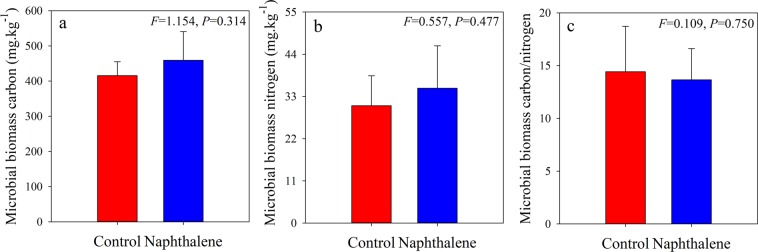
Soil microbial biomass carbon (**a**), soil microbial biomass nitrogen (**b**) and their ratio (**c**) for the control and naphthalene groups in a subalpine forest of western China. Values represent the means ± SEs (n = 5).

**Figure 2:**
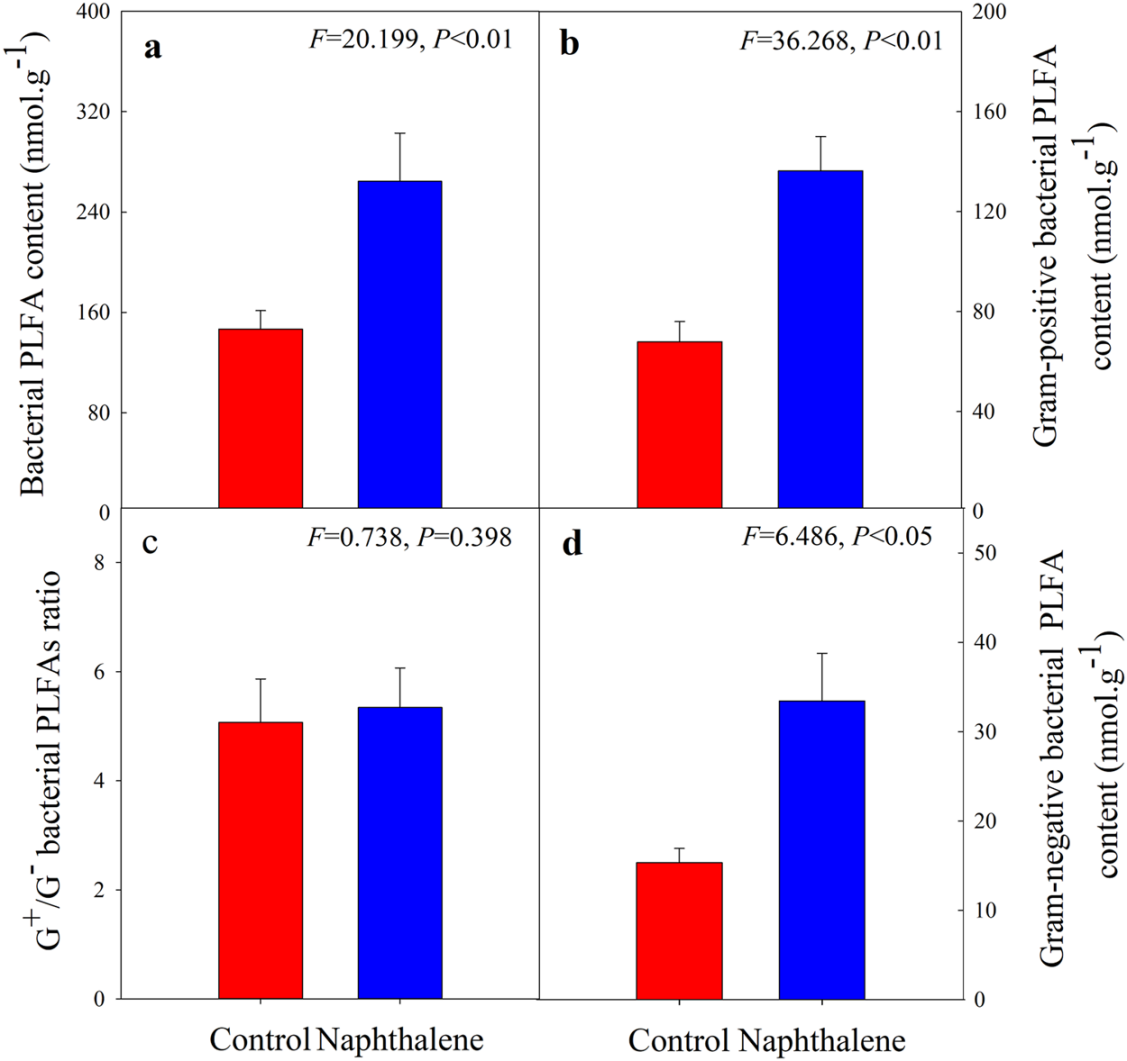
Soil phospholipid fatty acids for the control and naphthalene groups in a subalpine forest of western China. (**a**): total bacteria; (**b**): gram-positive bacteria; (**c**): G^+^/G^−^; (**d**): gram-negative bacteria. Values represent the means ± SEs (n = 5).

Naphthalene treatment slightly increased the concentrations of soil microbial biomass carbon (MBC) (Fig. 1a) and nitrogen (MBN) (Fig. 1b) but decreased the MBC/MBN ratio (Fig. 1c). The total bacterial, gram-positive (G^+^) bacterial, and gram-negative (G^−^) bacterial PLFA abundances were significantly increased in the naphthalene treatment (Fig. 2a,b,d), but the G^+^/G^−^ ratio did not change significantly (Fig. 2c).

### Sequencing results and diversity indices

A total of 543367 high-quality bacterial sequences were identified across all the samples. After normalization, the library contained 254240 reads, and the bacterial sequences were clustered into 1038 OTUs. The bacterial OTU numbers remained at a stable level among all treatments (Table 1, Fig. S1). All rarefaction curves tended to approach the saturation plateau, indicating that the data volume of sequenced reads was reasonable (Fig. S1). The good coverage values of 0.991 to 0.994 were observed for both the control and naphthalene groups (Table 1). Bacterial alpha diversity indices, including the abundance-based estimator (ACE), the Chao1 index, observed richness (Sobs), and Shannon-Wiener and Simpson’s diversity, exhibited no significant differences between the control and naphthalene treatments (*P* > 0.05; Table 1). This indicated that the naphthalene treatment did not significantly affect the diversity of the soil bacterial community.

**Table 1:** Sequence number, OTUs and alpha diversity indices in the control and naphthalene groups. Data are the means ± SEs (n = 5).

Groups	Sequence number	OTUs	ACE	Chao1	Sobs	Shannon	Simpson	Coverage
Control	53865 ± 3152a	994 ± 11a	1534 ± 15a	1553 ± 17a	1412 ± 14a	6.05 ± 0.03a	0.008 ± 0.001a	99.30%
Naphthalene	54808 ± 4226a	998 ± 26a	1528 ± 31a	1544 ± 33a	1410 ± 38a	5.99 ± 0.08a	0.010 ± 0.002a	99.30%

The coverage percentages, richness estimators (ACE and Chao1), and diversity indices (Shannon-Wiener and Simpson) were calculated. The same letters indicate no significant difference between the control and naphthalene groups (did not differ significantly at *P* < 0.05).

### Taxonomy and difference in composition of the bacterial community

The Venn diagram showed that all bacterial species at the phylum, genus, and OTU levels were shared between the control and naphthalene groups (Fig. S2). The community composition analysis was based on the phyla or genera shared by the two groups (at a 97% sequence similarity).

**Figure 3:**
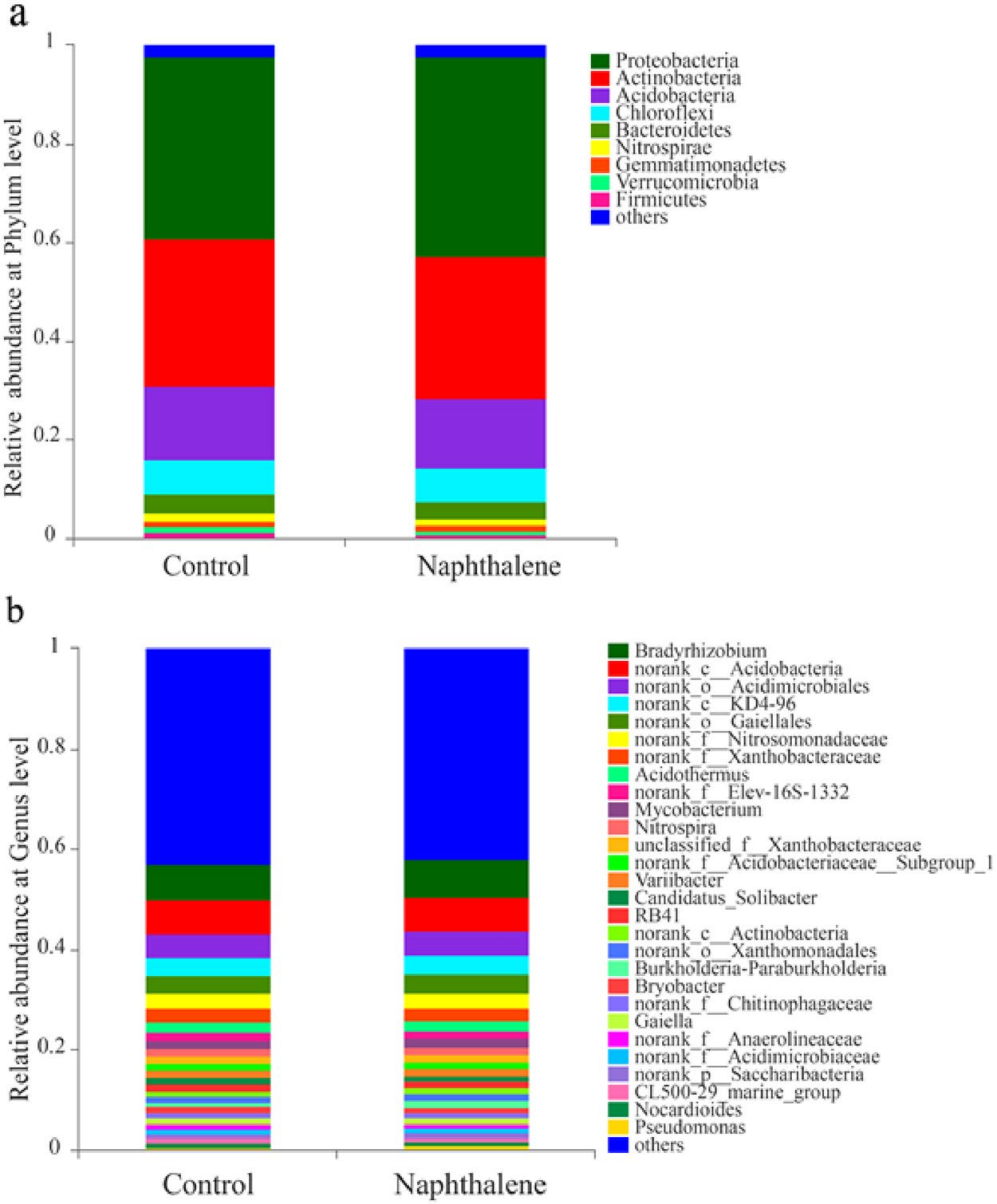
Bacterial community relative abundance analysis at the phylum (**a**) and genus (**b**) levels (relative abundance >1%; bacteria with relative abundances <1% were pooled in the ‘others’ category and sorted by total concentration). Data are the mean values of five samples for each group.

**Figure 4:**
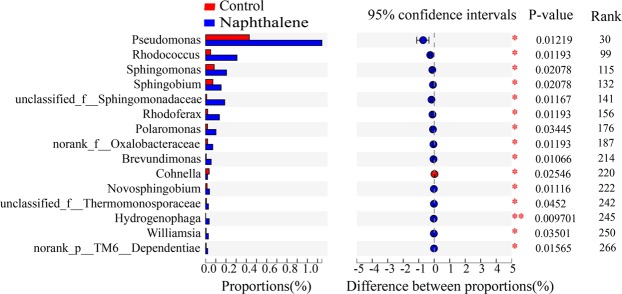
Comparison of the genera that differed significantly between the control and naphthalene treatment groups. The vertical axis represents the species names at the genus level, and each column corresponds to the species and represents the average relative abundance of the species in various groups (P value: *0.01 < P ≤ 0.05, **0.001 < P ≤ 0.01; Rank: the rank sum of the value).

Across all sites, bacterial communities were consistently dominated by globally distributed bacterial phyla: Proteobacteria (36.83–39.48%), Actinobacteria (28.21–29.19%), Acidobacteria (13.96–14.32%) and Chloroflexi (7.14–7.16%). In the community composition analysis, these four phyla constituted more than 87% of the total reads in the library (Fig. 3a). The naphthalene treatment did not alter the relative abundance of bacterial phyla in our study (Fig. S3; *P* > 0.05). At the genus level, 30 distinct groups were observed in all treatments (relative abundance greater than 1%) (Fig. 3b). *Bradyrhizobium* was the most abundant genus and accounted for approximately 7.3% of the total groups (Fig. 3b). The genera whose relative more than 1% did not significantly differ between the control and naphthalene groups (Fig. S4, *P* > 0.05), except for *Pseudomonas* (Fig. S4; *P* < 0.05). In addition, certain genera with a lower concentration (relative abundance less than 1%), such as *Rhodococcus*, *Sphingomonas*, and *Sphingobium*, exhibited significant differences between the control and naphthalene treatment groups (Fig. 4; *P* < 0.05). Most of these genera were much more abundant in the naphthalene treatment group than in the control group (Fig. 4).

**Figure 5:**
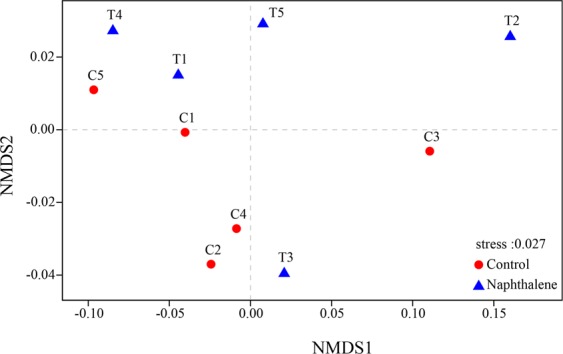
Sample sorting analysis. NMDS shows the differences in the bacterial communities according to the Bray-Curtis distance. If the stress value (in the bottom right corner) is less than 0.05, the result is well representative.

The composition of bacteria was further investigated with non-metric multidimensional scaling (NMDS) analysis (Fig. 5). The resolutions of NMDS suggested that the naphthalene treatment did not cause obvious separation in bacterial communities at the phylum level.

## Discussion

Naphthalene treatment is a practical approach used to suppress soil arthropods in the study area^19^, which may provide a feasible method of determining the functional roles of soil arthropods in these forests. However, one of the major criticisms of using naphthalene to exclude arthropods in field studies is the potential for effects on non-target organisms^17,18^. Thus, naphthalene was added to surface soil to inhibit the soil arthropods and determine whether the naphthalene treatment had non-target effects on soil microbial biomass, active bacterial abundance and the bacterial community. In general, the results showed that the soil arthropod suppression (based on individual density) approached 80% in this study (Fig. S5), which was similar to a previous study^14,18,20^. Moreover, the naphthalene treatment did not significantly change the MBC, MBN, MBC/MBN ratio or bacterial community structure but exerted substantial effects on the active bacterial community abundance.

Soil microorganisms play important and irreplaceable roles, such as in soil formation, soil fertility maintenance, material circulation and energy flow in forest ecosystems^21,22^. Bacteria are dominant microorganisms in alpine forest soils due to their relatively strong cold resistance^23,24^. In our study, the naphthalene treatment significantly increased the PLFA of the total bacteria, gram-positive (G^+^) bacteria, and gram-negative (G^−^) bacteria, which was consistent with a previous study^18^, indicating bacterial utilization of naphthalene-derived C. Chemical applications have been increasingly acknowledged to introduce exogenous nutrients, such as C, N and P, which provide an energy source for soil microbes^16^. Certain bacteria have been shown to use carbon and nitrogen from naphthalene as a substrate for growth^25,26^. The naphthalene treatment also significantly increased the abundance of some genera in our study (Fig. 4). *Pseudomonas* is a group of gram-negative bacteria that is well known for its strong ability to tolerate and degrade various aromatic pollutants^27,28^. A previous study investigated the fatty acid composition during naphthalene utilization in three strains of *Pseudomonas* sp. that express different naphthalene degradation abilities^29^. Their results showed that *Pseudomonas* sp. underwent changes in cell-derived fatty acids when grown with naphthalene as a carbon source, which may account for the significant increase in bacterial PLFA in our study. Similar results were obtained in another study^30^, in which changes in whole cell-derived fatty acids induced by benzene were observed in *Rhodococcus* sp. In addition, *Sphingomonas* sp. can utilize naphthalene as a sole source of carbon and energy for growth^31^. As a result, bacteria grow rapidly when they obtain sufficient exogenous C from naphthalene. However, the results of laboratory simulation experiments indicate that naphthalene treatment does not significantly affect total bacterial, gram-positive bacterial, or gram-negative bacterial PLFA^32^, which is inconsistent with the findings of field research. The limitations of the microcosm control experiment and the lack of aboveground vegetation may be two reasons for the differences in experimental results^18,32^.

The response of soil microbial biomass to the external environment can help determine soil quality^33^. The naphthalene treatment did not significantly affect the soil microbial biomass in our study, which was consistent with another study^20^. A previous study used two naphthalene treatments with different concentrations (100 g.m^−2^ and 200 g.m^−2^) to determine the change in MBC, and the result showed that the low-naphthalene treatment did not significantly affect the MBC^16^. Similarly, the G^+^/G^−^ bacterial ratios (Fig. 2c), NMDS analysis (Fig. 5) and alpha diversity indices (Table 1) all showed that the naphthalene treatment did not have a significant effect on the diversity and structure of the bacterial community. On the one hand, soil bacteria can stabilize the community structure by self-regulation. Soil bacteria may die due to low tolerance to low-temperature stress^34^. However, carbon and nutrients released upon microbial death can be rapidly accumulated and increased^35,36^. The effective matrix that is released can be used directly or indirectly by living organisms in the soil, resulting in an increased number of microorganisms^32,37^. On the other hand, soil bacterial populations are regulated by interactions with soil fauna^7^. Soil bacteria may increase due to a decrease in the bacterial predator (soil arthropod)^1,2^. However, an increase in the abundance of soil bacteria could lead to competition among populations for nutrients and space^9,10^. As a result, the soil bacterial community may remain stable via different mechanisms of regulation.

## Conclusion

In this field experiment, naphthalene was added to the forest soil surface to assess the non-target effects of naphthalene on the soil bacterial community in the subalpine forests of western China. Overall, the naphthalene treatment led to an increase in bacterial PLFA abundance, which might have been caused by the utilization of naphthalene. Soil microbial biomass and bacterial community structure and diversity were not significantly affected by naphthalene application due to the various regulatory mechanisms of the soil bacterial community. Our study demonstrated that naphthalene treatment is a proper method of manipulating soil arthropods in field studies and may promote soil bacterial growth. Whether the changes differ in other regions is unclear. Thus, a pre-experiment should be performed before using naphthalene to expel soil fauna.

## Materials and Methods

### Study site

The field experiment was conducted in a secondary fir (*Abies faxoniana*) forest at the Long-term Research Station of Alpine Forest Ecosystems on the eastern Tibetan Plateau, China (31°18′N, 102°56′E, 3023 m *a.s.l*.). A forest that had been formed by seeding clear-cut lands since the 1960s was used as the study site at the station. The annual mean temperature and precipitation at the site are 2.7 °C and 850 mm, respectively^19^. The tree canopy was dominated by fir with an age of 60 years, and the average tree canopy coverage, tree height and diameter at breast height (DBH) were 0.7, 17 m and 24 cm, respectively^19^. The dominant understory shrub species were *Salix paraplesia*, *Fargesia nitida*, *Rhododendron lapponicum*, *Berberis sargentiana*, *Sorbus rufopilosa, Rosa sweginzowii* and *Hippophae rhamnoides*^19^. The herbs included *Cacalia auriculata*, *Cystopteris montana*, *Carex* spp. and *Cyperus* spp^19^. The soil was classified as a Cambic Umbrisol per the IUSS Working Group, and more details on soil chemical properties can be found in^19^.

### Experimental design

A sample area (100 m × 100 m) was established at an altitude of approximately 3000 metres in the secondary fir forest. Each treatment had five plots or replicates (5 m × 5 m each), with ≥10 m between each plot^19^. Within each plot, there were four subplots (2 m × 2 m each), with one treated with naphthalene, one serving as the control, and the remaining two treated by another experimental design (no effect in this study)^19^. Each subplot spanned 1 m, and a PVC fence (2 m × 2 m × 10 cm) was inserted into the ground to a 5-cm depth^19^. The naphthalene addition started in early October 2015, and the naphthalene treatment subplots received the equivalent of 100 g.m^−2^ naphthalene every month for the full duration of the experiment^16–19^.

### Soil sampling and soil arthropod extraction

Soil sampling was performed on 23 October 2017. Specifically, an intact square soil core (20 cm × 25 cm) was collected to a depth of 10 cm from the control and naphthalene treatment subplots to extract soil arthropods^19^. Soil arthropods were extracted from the intact soil cores using the Tullgren funnel (mesh size: 4.00 mm) method over a 48-h period^19^. The naphthalene treatment significantly reduced both individuals and groups of soil arthropods (Fig. S5), and more details on soil arthropod suppression can be found in^19^. Additionally, for each subplot, five soil cores (approximately 100 g each) were collected using a soil auger (15-cm depth and 5-cm diameter) and mixed into one composite sample after removing visible debris and fresh litter^19^. Soil samples (n = 10) were sent to the laboratory and stored in a freezer within 24 h. Soil samples were passed through a 2.0-mm sieve and divided into two subsamples. One was kept in a freezer at −70 °C for soil microbial analysis, and the other was kept at 4 °C for chemical analysis within one week.

### Microbial biomass and PLFA analysis

The soil MBC and MBN were determined by the chloroform fumigation extraction method with a conversion factor of 0.45 for MBC and 0.54 for MBN^38,39^. PLFA are a good indicator of living organisms and can be used to adequately detect rapid changes in soil microbial communities because they are essential components of the membranes of all living microbes and degrade rapidly with cell death^40,41^. The PLFA concentrations in three freeze-dried subsamples were determined with the methods of a previous study, with minor modifications^19,40,42^. Briefly, total lipids were extracted from 1 g of slightly thawed soil via a one-phase extraction technique using phosphate buffer, methanol and chloroform in a 0.8:2:1 (v/v/v) ratio^19^. After adding an internal standard (19:0), PLFA were converted to fatty acid methyl esters (FAMEs) by alkaline methanolysis and were then extracted by mild alkaline methanolysis^19^. The samples were dissolved in hexane and analysed using a SHIMADZU gas chromatograph equipped with a mass spectrometer (QP2010-Ultra) and a GC column (Cat No. 13623) and controlled by an operation system with reference to the standards^40,42^. Phospholipid FAME standards were obtained from Supelco (Bacterial Acid Methyl Ester Mix, 47080-U; 37 Component Fatty Acid Methyl Ester Mix, CRM47885)^42^. The internal standard of methyl nonadecanoate (C19:0) was used to calculate the PLFA contents^42^. The details of the gas chromatography-mass spectrometry (GC-MS) conditions and the general bacterial markers were described previously^19,43–45^. Gram-positive, gram-negative and general bacterial markers were summed to determine the total bacteria^43–45^.

### DNA extraction, PCR amplification and Illumina MiSeq sequencing

Microbial DNA was extracted in triplicate from each mixed soil sample using an E.Z.N.A.^®^ soil DNA Kit (Omega Bio Inc., Norcross, GA, USA) according to the manufacturer’s protocols. The final DNA concentration and purification were determined by a NanoDrop 2000 UV-vis spectrophotometer (Thermo Scientific Inc., Wilmington, DE, USA), and DNA quality was checked by 1% agarose gel electrophoresis.

The V3-V4 hypervariable region of the bacterial 16S rRNA gene was amplified using the primers 338 F/806R^22^ by a thermocycler Geneamp PCR system 9700 (Applied Biosystems Inc., Foster City, CA, USA). PCRs were conducted using the following programme: 3 min of denaturation at 95 °C; 27 cycles of 30 s at 95 °C, 30 s of annealing at 55 °C, and 45 s of elongation at 72 °C, and a final extension at 72 °C for 10 min. PCR was performed in triplicate using a 20-μL mixture containing 4 μL of 5× FastPfu buffer, 2 μL of 2.5 mM dNTPs, 0.8 μL of each primer (5 μM), 0.4 μL of FastPfu polymerase and 10 ng of template DNA. The resulting PCR products were extracted from a 2% agarose gel, further purified using an AxyPrep DNA Gel Extraction Kit (Axygen Biosciences, Union City, CA, USA) and quantified using a QuantiFluor™-ST fluorimeter (Promega, USA) according to the manufacturer’s protocol. The purified amplicons were merged in equimolar amounts and paired-end sequenced (2 × 300) on an Illumina MiSeq platform (Illumina, San Diego, CA, USA) according to the standard protocols by Majorbio Bio-Pharm Technology Co. Ltd. (Shanghai, China).

### Processing of sequence data

Raw FASTQ files were demultiplexed and quality-filtered by Trimmomatic and merged by FLASH with the following criteria: (1) the reads were truncated at any site receiving an average quality score <20 over a 50-bp sliding window. (2) Primers were exactly matched, allowing 2-nucleotide mismatching, and reads containing ambiguous bases were removed. (3) Sequences whose overlap was longer than 10 bp were merged according to their overlap sequence^22^.

OTUs were clustered with a 97% similarity cut-off using UPARSE (version 7.1 http://drive5.com/uparse/), and chimeric sequences were identified and removed using UCHIME^46^. The taxonomy of each 16S rRNA gene sequence was analysed using the ribosomal database project (RDP) classifier algorithm (http://rdp.cme.msu.edu/) against the Silva (SSU123) 16S rRNA database using a confidence threshold of 70%^47^.

### Statistical analysis

Alpha diversity metrics, including the Shannon-Wiener index, Simpson’s diversity index, Sobs, the ACE estimator, and the Chao1 estimator, were calculated using the “diversity” and “richness” functions in the vegan package of R software. The bacterial community structure was visualized by NMDS ordinations using the vegan package in R software. In addition to the statistical algorithms and mapping software used in the above biological information analysis, figures were drawn using SigmaPlot 12.5, and statistical tests were performed using IBM SPSS Statistics 20.0 (SPSS Inc., Chicago, IL, USA). Independent t-tests were used to examine the differences in microbial biomass, PLFAs and bacterial community diversity indices between the control and treatment groups. Statistical tests were considered significant at *P* < 0.05.

## Supplementary information

Supplementary information

## Data Availability

The data presented in this paper can be found in the supporting information.
