# Supplementary material for: Non-target Effects of Naphthalene on the Soil Microbial Biomass and Bacterial Communities in the Subalpine Forests of Western China
Source: Sci Rep. 2019 Jul 8;9:9811. doi: 10.1038/s41598-019-46394-3 (PMC6614484; doi:10.1038/s41598-019-46394-3)
Supplement: Supplementary file 1 — Supplementary information [file 41598_2019_46394_MOESM1_ESM.pdf]

SUPPLEMENTARY INFORMATION

Title:

Non-target Effects of Naphthalene on the Soil Microbial Biomass and Bacterial Communities in the Subalpine Forests of Western China

Authors:

Liying Lan [1,2], Fan Yang [1,2], Li Zhang [1,2], Wanqin Yang [1,2], Fuzhong Wu [1,2], Zhenfeng Xu [1,2], Yang Liu [1,2], Kai Yue [1,2], Xiangyin Ni [1,2], Han Li [1,2], Shu Liao[1,2], Yuwei Liu [1,2], Ya Chen [1,2] & Bo Tan [1,2*]

Affiliation:

1 Institute of Ecology & Forestry, Sichuan Agricultural University, Forestry Ecological Engineering in Upper Reaches of Yangtze River Key Laboratory of Sichuan Province, Alpine Forest Ecosystem Research Station, Soil and Water Conservation and Desertification Control Key Laboratory of Sichuan Province, Chengdu 611130, China

2 Collaborative Innovation Center of Ecological Security in the Upper Reaches of Yangtze River, Chengdu 611130, China

Liying Lan, Fan Yang and Li Zhang contributed equally to this work.

Corresponding authors (*):

B. Tan (E-mail address: bobotan1984@163.com)

Tel.: 86-28-86290957

Postal address: 211 Huimin Road, Wenjiang, Chengdu 611130, Sichuan, China

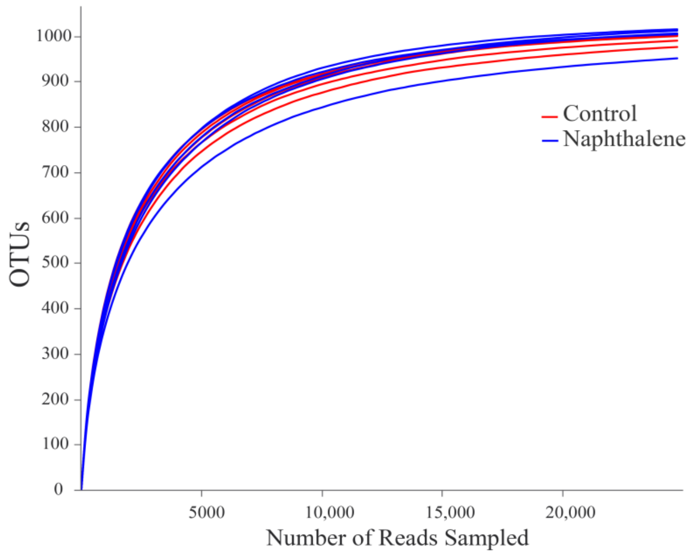

Figure S1 Rarefaction curves of the number of OTUs at 97% sequence similarity for every sample.

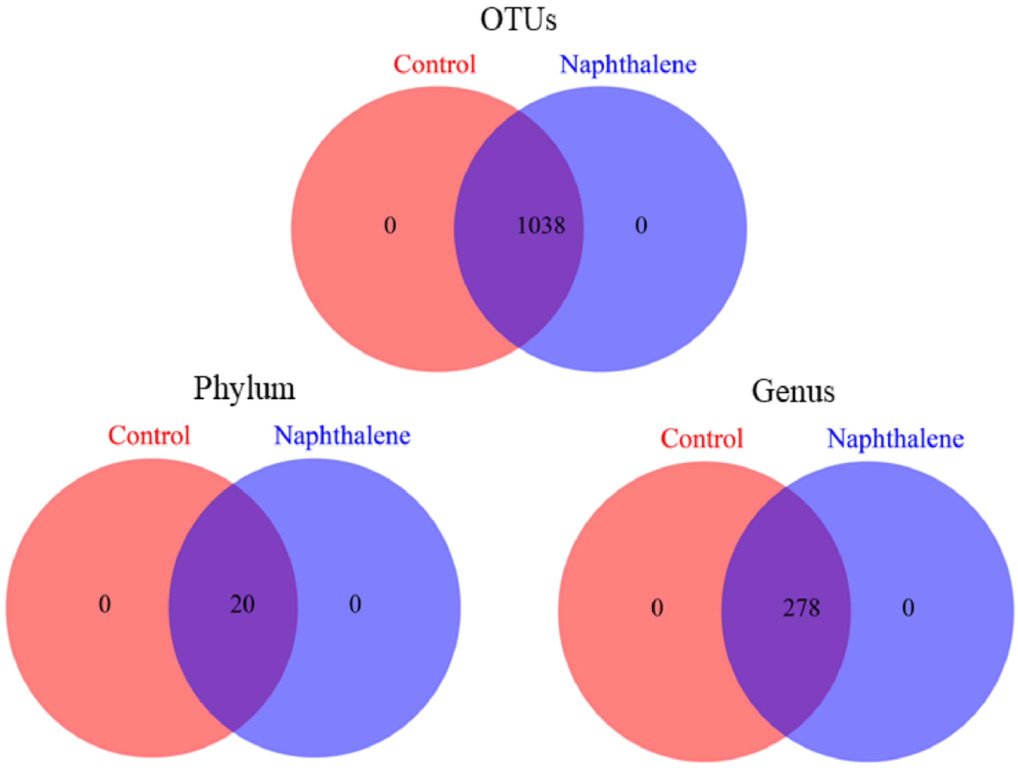

Figure S2 Venn diagram between the control and naphthalene groups at the OTU, phylum and genus levels (n = 5).

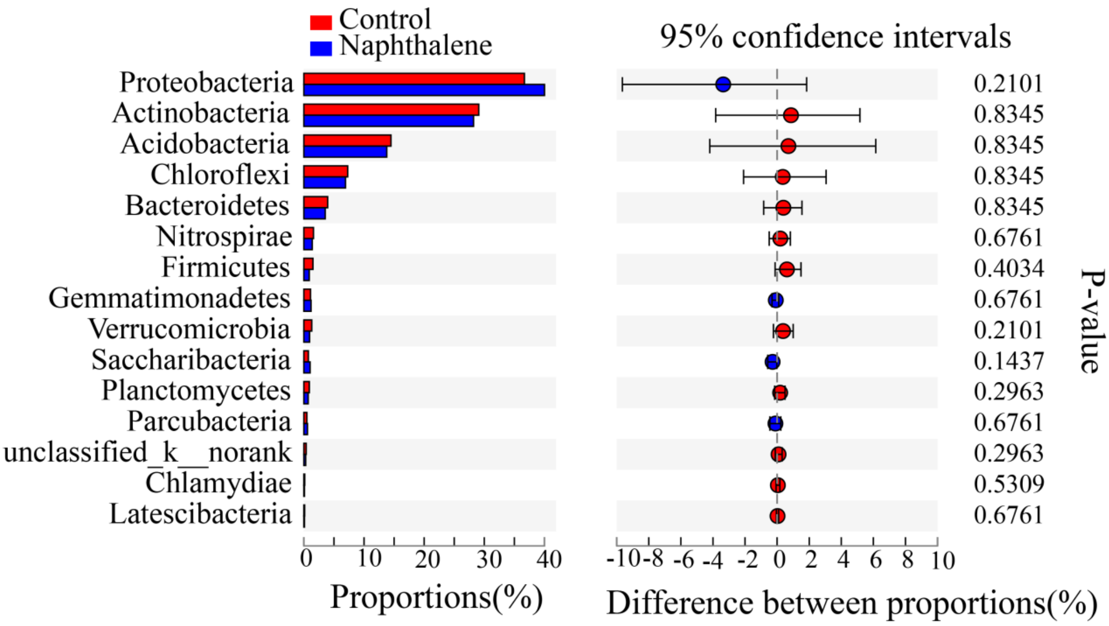

Figure S3 Bar plot of a Wilcoxon rank-sum test at the phylum level.

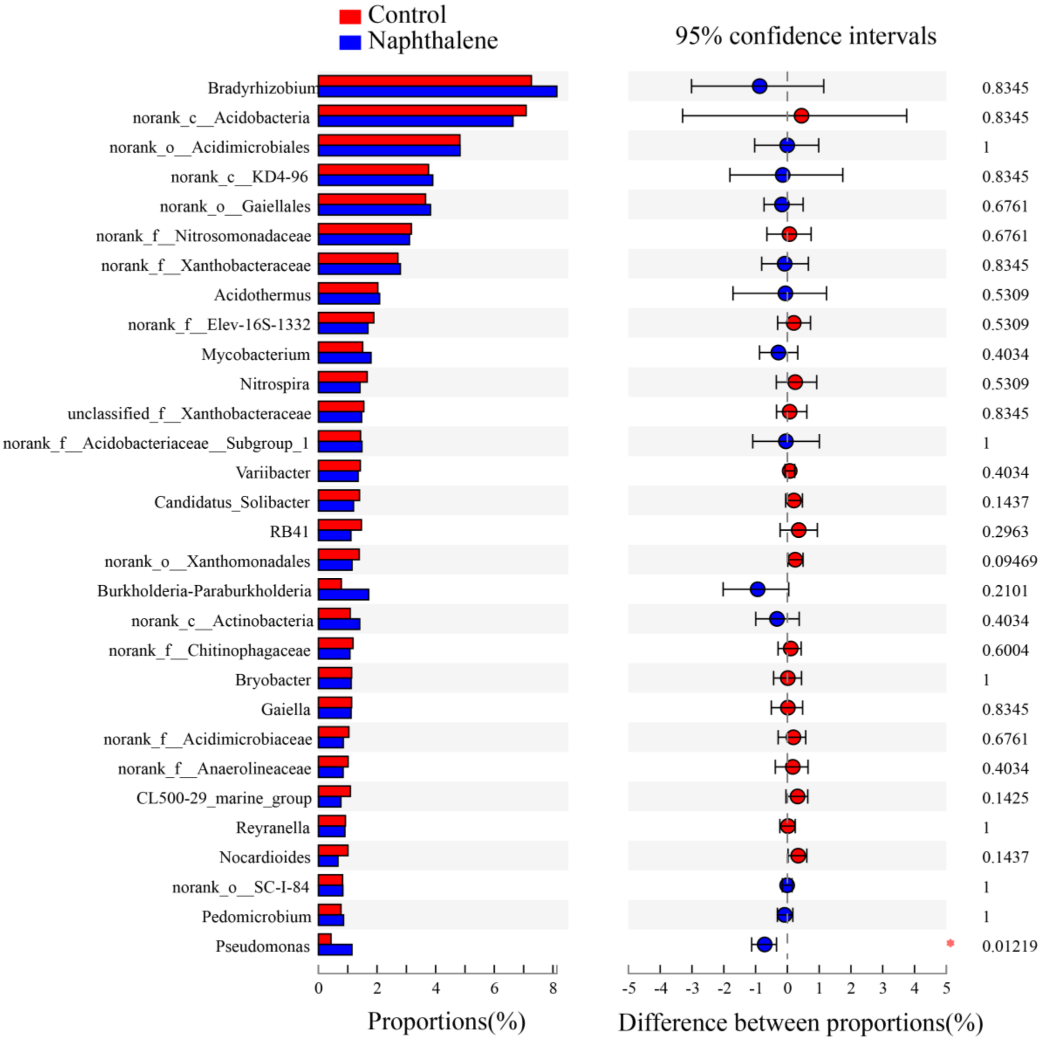

Figure S4 Bar plot of a Wilcoxon rank-sum test at the genus level. The genera are the top 30 most abundant genera in the community (ranked by the sum of means).

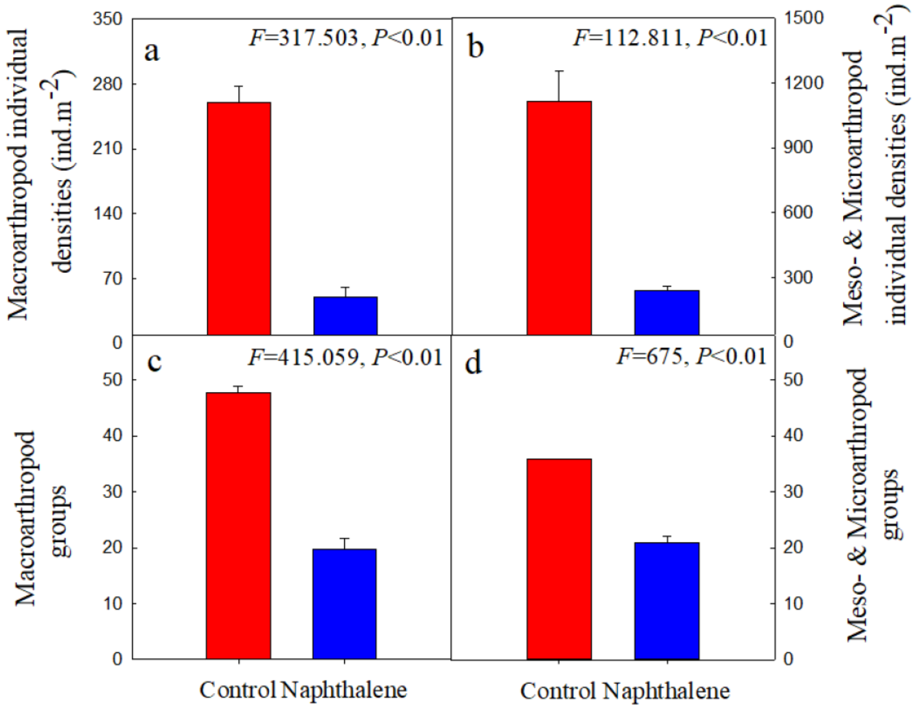

Fig. S5 Effectiveness of naphthalene treatment for inhibiting soil arthropods in a subalpine forest of western China. The upper right corner of each diagram shows the statistics (the control and naphthalene groups differed significantly at P<0.05). Values represent the means ±SEs (n=5).

For more details on the suppression of the soil fauna community in this study, see https://doi.org/10.1038/s41598-019-39603-6.
